# A second triclinic polymorph of azimsulfuron

**DOI:** 10.1107/S2056989016014845

**Published:** 2016-09-23

**Authors:** Eunjin Kwon, Jineun Kim, Hyunjin Park, Tae Ho Kim

**Affiliations:** a Department of Chemistry (BK21 plus) and Research Institute of Natural Sciences, Gyeongsang National University, Jinju 52828, Republic of Korea

**Keywords:** crystal structure, sulfonyl­urea herbicide, azimsulfuron, dimorphism

## Abstract

The title compound, C_13_H_16_N_10_O_5_S (systematic name: 1-(4,6-di­meth­oxypyrimidin-2-yl)-3-{[1-methyl-4-(2-methyl-2*H*-tetra­zol-5-yl)pyrazol-5-yl]sulfonyl}urea), is a second triclinic polymorph of this crystal [for the other, see: Jeon *et al.*, (2015 ▸). *Acta Cryst*. E**71**, o470–o471]. There are two mol­ecules, *A* and *B*, in the asymmetric unit; the dihedral angles between the pyrazole ring and the tetra­zole and di­meth­oxy­pyrimidine ring planes are 72.84 (10) and 37.24 (14)°, respectively (mol­ecule *A*) and 84.38 (9) and 26.09 (15)°, respectively (mol­ecule *B*). Each mol­ecule features an intra­molecular N—H⋯N hydrogen bond. In the crystal, aromatic π–π stacking inter­actions [centroid–centroid separations = 3.9871 (16), 3.4487 (14) and 3.5455 (16) Å] link the mol­ecules into [001] chains. In addition, N—H⋯N, N—H⋯O, C—H⋯O and C—H⋯N hydrogen bonds occur, forming a three-dimensional architecture. We propose that the dimorphism results from differences in conformations and packing owing to different inter­molecular inter­actions, especially aromatic π–π stacking.

## Abstract 2

The title compound crystallizes with two mol­ecules in the asymmetric unit, each mol­ecule adopting a ‘boxing-glove’ shape.

## Chemical context   

Sulfonyl­urea herbicides are well known as being highly beneficial for controlling undesirable vegetation in agronomic­ally desirable crops including corn and cereals such as wheat and barley. Azimsulfuron is a recently introduced highly selective sulfonyl­urea herbicide (Valle *et al.*, 2006 ▸) and has been found to be particularly useful as a post-emergent herbicide for weed control in rice paddies and suppression of barnyard grass in rice (Venkatesh *et al.*, 2016 ▸). The crystal structure of azimsulfuron (dimorph I) has already been reported in our previous study (Jeon *et al.*, 2015 ▸). We now report the crystal structure of a second triclinic polymorph, grown under different conditions, as observed for other systems (Schmidt & Jansen, 2012 ▸; Ebenezer & Mu­thiah, 2010 ▸).
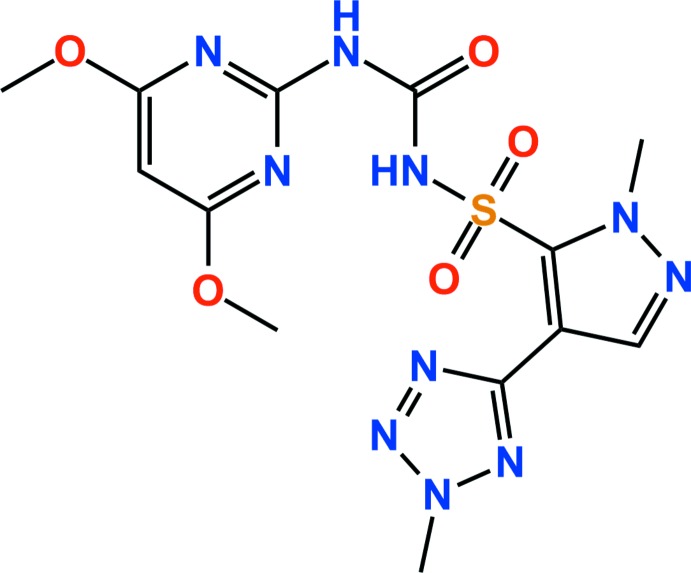


## Structural commentary   

**Figure 1:**
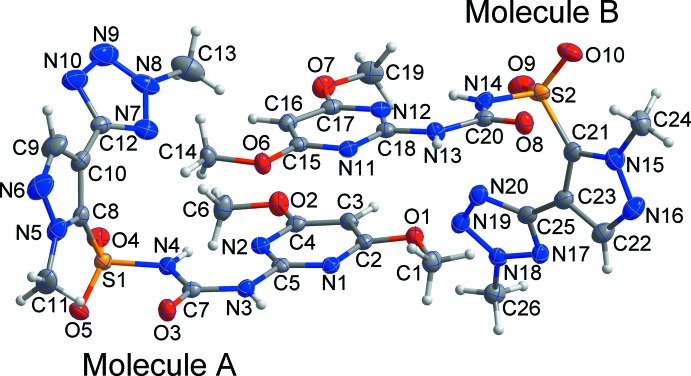
The asymmetric unit of the title compound, with displacement ellipsoids drawn at the 50% probability level. H atoms are shown as small spheres of arbitrary radius.

The asymmetric unit of the new dimorph II (Fig. 1 ▸), consists of two independent mol­ecules, *A* and *B*, which are curved and form a ‘boxing glove’ shape around the pyrazole ring. The dihedral angles between the pyrazole ring and the tetra­zole and di­meth­oxy­pyrimidine ring planes are 72.84 (10) and 37.24 (14)°, respectively, in mol­ecule *A* and 84.38 (9) and 26.09 (15)°, respectively, in mol­ecule *B*. All bond lengths and bond angles are normal and comparable to those observed in similar crystal structures (Chopra *et al.*, 2004 ▸; Kwon *et al.*, 2015 ▸). Each mol­ecule features an intra­molecular N—H⋯N hydrogen bond (Table 1 ▸), which closes an *S*(6) ring.

## Supra­molecular features   

**Figure 2:**
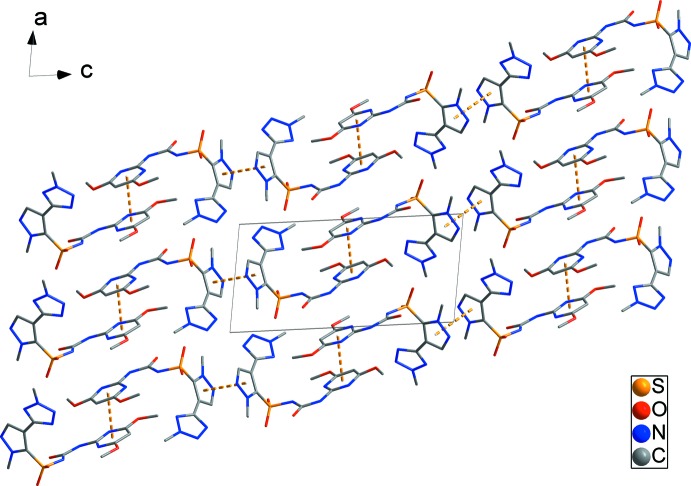
The crystal packing, viewed along the *b* axis. H atoms have been omitted for clarity.

In the crystal, aromatic π–π stacking inter­actions, *Cg*1⋯*Cg*1^iii^ = 3.9817 (16), *Cg*3⋯*Cg*6^vii^ = 3.4487 (14) and *Cg*4⋯*Cg*4^viii^ = 3.5455 (16) Å occur [*Cg*1, *Cg*3, *Cg*4 and *Cg*6 are the centroids of the N5/N6/C8–C10, N1/N2/C2–C5, N15/N16/C21–C23 and N11/N12/C15–C18 rings, respectively; symmetry codes: (vii) *x*, *y*, *z*, (viii) −*x* + 2, −*y*, −*z* + 2]. Together, these link adjacent mol­ecules, forming chains propagating along the *c* axis. (Fig. 2 ▸). In addition, there are N—H⋯N, N—H⋯O, C—H⋯O and C—H⋯N hydrogen bonds in dimorph II (Table 1 ▸), which generate a three-dimensional architecture.

The previous dimorph has only one π–π inter­action between the tetra­zole rings of neighboring mol­ecules, while the present dimorph has three π–π inter­actions between di­meth­oxy­pyrimidine rings in the asymmetric unit, and between the pyrazole rings of neighboring *A* or *B* mol­ecules.

## Synthesis and crystallization   

In the previous report, crystals were obtained by using CH_3_CN solvent, whereas colourless needles of the title polymorph were prepared by slow evaporation of a methanol solution.

## Refinement   

Crystal data, data collection and structure refinement details are summarized in Table 2 ▸. All H atoms were positioned geometrically and refined using a riding model with *d*(N—H) = 0.88 Å, *U*_iso_ = 1.2*U*_eq_(C) for N—H groups, *d*(C—H) = 0.88 Å, *U*_iso_ = 1.2*U*_eq_(C) for C*sp* ^2^—H and *d*(C—H) = 0.98 Å, *U*_iso_ = 1.5*U*_eq_(C) for methyl groups.

## Supplementary Material

Crystal structure: contains datablock(s) I, New_Global_Publ_Block. DOI: 10.1107/S2056989016014845/hb7615sup1.cif

Structure factors: contains datablock(s) I. DOI: 10.1107/S2056989016014845/hb7615Isup2.hkl

Supporting information file. DOI: 10.1107/S2056989016014845/hb7615Isup3.cml

CCDC reference: 1505302

Additional supporting information: crystallographic information; 3D view; checkCIF report

## Figures and Tables

**Table 1:** Hydrogen-bond geometry (Å, °)

*D*—H⋯*A*	*D*—H	H⋯*A*	*D*⋯*A*	*D*—H⋯*A*
N3—H3*N*⋯N11^i^	0.88	2.65	3.494 (3)	162
N4—H4*N*⋯N2	0.88	1.89	2.611 (3)	138
N13—H13*N*⋯O3^i^	0.88	2.04	2.844 (2)	151
N14—H14*N*⋯N12	0.88	1.95	2.636 (3)	134
C1—H1*C*⋯O8^ii^	0.98	2.58	3.559 (3)	178
C11—H11*B*⋯O3	0.98	2.38	3.236 (3)	146
C11—H11*B*⋯O8^i^	0.98	2.57	3.278 (3)	129
C11—H11*C*⋯N10^iii^	0.98	2.58	3.290 (4)	130
C13—H13*A*⋯O9^iv^	0.98	2.40	3.374 (4)	175
C14—H14*A*⋯O5^v^	0.98	2.39	3.335 (3)	162
C22—H22⋯N17^vi^	0.95	2.57	3.435 (3)	152
C24—H24*B*⋯O8	0.98	2.33	3.080 (3)	133
C26—H26*B*⋯N16^vi^	0.98	2.52	3.376 (3)	146

Symmetry codes: (i) 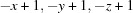; (ii) 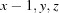; (iii) 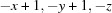; (iv) 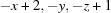; (v) 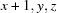; (vi) 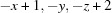.

**Table 2:** Experimental details

Crystal data	
Chemical formula	C_13_H_16_N_10_O_5_S
*M*_r_	424.42
Crystal system, space group	Triclinic, *P* 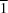
Temperature (K)	173
*a*, *b*, *c* (Å)	7.6451 (2), 15.1102 (4), 17.0314 (5)
α, β, γ (°)	67.1562 (18), 80.5936 (17), 84.0996 (17)
*V* (Å^3^)	1787.08 (9)
*Z*	4
Radiation type	Mo *K*α
μ (mm^−1^)	0.24
Crystal size (mm)	0.30 × 0.04 × 0.04

Data collection	
Diffractometer	Bruker APEXII CCD
Absorption correction	Multi-scan (*SADABS*; Bruker, 2014 ▸)
*T*_min_, *T*_max_	0.686, 0.746
No. of measured, independent and observed [*I* > 2σ(*I*)] reflections	25718, 6265, 4741
*R*_int_	0.060
(sin θ/λ)_max_ (Å^−1^)	0.595

Refinement	
*R*[*F* ^2^ > 2σ(*F* ^2^)], *wR*(*F* ^2^), *S*	0.045, 0.114, 1.07
No. of reflections	6265
No. of parameters	531
H-atom treatment	H-atom parameters constrained
Δρ_max_, Δρ_min_ (e Å^−3^)	0.38, −0.39

Computer programs: *APEX2* and *SAINT* (Bruker, 2014 ▸), *SHELXS97* and *SHELXTL* (Sheldrick, 2008 ▸), *SHELXL2014* (Sheldrick, 2015 ▸), *DIAMOND* (Brandenburg, 2010 ▸) and *publCIF* (Westrip, 2010 ▸).

## supplementary crystallographic information

### Crystal data

**Table:**

C_13_H_16_N_10_O_5_S	*Z* = 4
*M**_r_* = 424.42	*F*(000) = 880
Triclinic, *P*1	*D*_x_ = 1.577 Mg m^−^^3^
*a* = 7.6451 (2) Å	Mo *K*α radiation, λ = 0.71073 Å
*b* = 15.1102 (4) Å	Cell parameters from 3336 reflections
*c* = 17.0314 (5) Å	θ = 2.3–24.2°
α = 67.1562 (18)°	µ = 0.24 mm^−^^1^
β = 80.5936 (17)°	*T* = 173 K
γ = 84.0996 (17)°	Needle, colourless
*V* = 1787.08 (9) Å^3^	0.30 × 0.04 × 0.04 mm

### Data collection

**Table:**

Bruker APEXII CCD diffractometer	4741 reflections with *I* > 2σ(*I*)
φ and ω scans	*R*_int_ = 0.060
Absorption correction: multi-scan (SADABS; Bruker, 2014)	θ_max_ = 25.0°, θ_min_ = 1.5°
*T*_min_ = 0.686, *T*_max_ = 0.746	*h* = −8→9
25718 measured reflections	*k* = −17→17
6265 independent reflections	*l* = −20→20

### Refinement

**Table:**

Refinement on *F*^2^	0 restraints
Least-squares matrix: full	Hydrogen site location: inferred from neighbouring sites
*R*[*F*^2^ > 2σ(*F*^2^)] = 0.045	H-atom parameters constrained
*wR*(*F*^2^) = 0.114	*w* = 1/[σ^2^(*F*_o_^2^) + (0.0457*P*)^2^ + 0.2151*P*] where *P* = (*F*_o_^2^ + 2*F*_c_^2^)/3
*S* = 1.07	(Δ/σ)_max_ < 0.001
6265 reflections	Δρ_max_ = 0.38 e Å^−^^3^
531 parameters	Δρ_min_ = −0.39 e Å^−^^3^

### Special details

**Table:**

Geometry. All esds (except the esd in the dihedral angle between two l.s. planes) are estimated using the full covariance matrix. The cell esds are taken into account individually in the estimation of esds in distances, angles and torsion angles; correlations between esds in cell parameters are only used when they are defined by crystal symmetry. An approximate (isotropic) treatment of cell esds is used for estimating esds involving l.s. planes.

### Fractional atomic coordinates and isotropic or equivalent isotropic displacement parameters (Å^2^)

**Table:**

	*x*	*y*	*z*	*U*_iso_*/*U*_eq_	
S1	0.29659 (8)	0.39202 (4)	0.20930 (4)	0.02210 (17)	
S2	1.16721 (9)	0.10185 (4)	0.78289 (4)	0.02695 (18)	
O1	0.6095 (2)	0.23247 (11)	0.66818 (10)	0.0267 (4)	
O2	0.5902 (2)	0.10067 (11)	0.46454 (10)	0.0295 (4)	
O3	0.2038 (2)	0.50940 (11)	0.31822 (10)	0.0260 (4)	
O4	0.3602 (2)	0.30534 (11)	0.19780 (11)	0.0288 (4)	
O5	0.1186 (2)	0.42651 (12)	0.19829 (11)	0.0294 (4)	
O6	0.7598 (2)	0.48627 (11)	0.37801 (10)	0.0260 (4)	
O7	1.0204 (2)	0.17015 (12)	0.45730 (11)	0.0315 (4)	
O8	1.0811 (2)	0.30423 (11)	0.75749 (11)	0.0285 (4)	
O9	1.1625 (2)	0.01909 (11)	0.76253 (12)	0.0346 (5)	
O10	1.3265 (2)	0.12288 (12)	0.80359 (12)	0.0360 (5)	
N1	0.4586 (3)	0.31656 (13)	0.55273 (12)	0.0217 (5)	
N2	0.4580 (3)	0.25049 (13)	0.44580 (12)	0.0214 (5)	
N3	0.3252 (3)	0.40128 (13)	0.43281 (12)	0.0210 (5)	
H3N	0.2896	0.4405	0.4598	0.025*	
N4	0.3313 (3)	0.37078 (13)	0.30805 (12)	0.0212 (5)	
H4N	0.3867	0.3161	0.3356	0.025*	
N5	0.3879 (3)	0.57663 (15)	0.10476 (13)	0.0327 (6)	
N6	0.5224 (4)	0.62778 (16)	0.05092 (15)	0.0458 (7)	
N7	0.7561 (3)	0.31962 (15)	0.20532 (14)	0.0311 (5)	
N8	0.8972 (3)	0.26823 (15)	0.18460 (16)	0.0357 (6)	
N9	0.9657 (4)	0.3035 (2)	0.10352 (18)	0.0519 (7)	
N10	0.8695 (3)	0.3815 (2)	0.06687 (16)	0.0466 (7)	
N11	0.8673 (3)	0.41362 (13)	0.50271 (12)	0.0219 (5)	
N12	1.0024 (3)	0.25495 (13)	0.54475 (12)	0.0215 (5)	
N13	0.9852 (3)	0.34673 (13)	0.62868 (12)	0.0218 (5)	
H13N	0.9551	0.4035	0.6311	0.026*	
N14	1.1044 (3)	0.19100 (13)	0.69852 (12)	0.0242 (5)	
H14N	1.0987	0.1805	0.6517	0.029*	
N15	1.0132 (3)	0.13158 (14)	0.92857 (13)	0.0293 (5)	
N16	0.8644 (3)	0.11937 (15)	0.98511 (14)	0.0350 (6)	
N17	0.6174 (3)	−0.04932 (14)	0.89155 (12)	0.0247 (5)	
N18	0.5689 (3)	−0.07811 (14)	0.83420 (13)	0.0241 (5)	
N19	0.6566 (3)	−0.03852 (15)	0.75612 (13)	0.0298 (5)	
N20	0.7690 (3)	0.01978 (15)	0.76081 (13)	0.0295 (5)	
C1	0.5501 (4)	0.30726 (18)	0.70047 (16)	0.0298 (6)	
H1A	0.5859	0.3696	0.6566	0.045*	
H1B	0.6036	0.2952	0.7524	0.045*	
H1C	0.4206	0.3078	0.7143	0.045*	
C2	0.5514 (3)	0.23781 (16)	0.59675 (15)	0.0218 (6)	
C3	0.5963 (3)	0.16081 (16)	0.57120 (15)	0.0229 (6)	
H3	0.6570	0.1042	0.6051	0.028*	
C4	0.5473 (3)	0.17177 (16)	0.49379 (15)	0.0224 (6)	
C5	0.4184 (3)	0.31774 (16)	0.47966 (15)	0.0200 (5)	
C6	0.5328 (4)	0.11182 (18)	0.38456 (16)	0.0336 (7)	
H6A	0.4032	0.1201	0.3896	0.050*	
H6B	0.5705	0.0545	0.3715	0.050*	
H6C	0.5858	0.1684	0.3383	0.050*	
C7	0.2806 (3)	0.43161 (16)	0.35078 (15)	0.0200 (5)	
C8	0.4391 (4)	0.48225 (17)	0.14203 (15)	0.0241 (6)	
C9	0.6598 (4)	0.5660 (2)	0.05491 (18)	0.0427 (8)	
H9	0.7736	0.5832	0.0230	0.051*	
C10	0.6187 (4)	0.47261 (18)	0.11140 (15)	0.0281 (6)	
C11	0.2159 (4)	0.62701 (19)	0.11317 (18)	0.0393 (8)	
H11A	0.2286	0.6964	0.0824	0.059*	
H11B	0.1741	0.6133	0.1741	0.059*	
H11C	0.1298	0.6050	0.0887	0.059*	
C12	0.7435 (3)	0.38992 (18)	0.12941 (16)	0.0272 (6)	
C13	0.9717 (4)	0.1819 (2)	0.2470 (2)	0.0522 (9)	
H13A	0.9361	0.1250	0.2404	0.078*	
H13B	0.9277	0.1791	0.3053	0.078*	
H13C	1.1015	0.1837	0.2374	0.078*	
C14	0.7391 (4)	0.49082 (18)	0.29395 (15)	0.0278 (6)	
H14A	0.8562	0.4869	0.2617	0.042*	
H14B	0.6774	0.5516	0.2631	0.042*	
H14C	0.6697	0.4370	0.2998	0.042*	
C15	0.8417 (3)	0.40702 (16)	0.42896 (15)	0.0217 (6)	
C16	0.8940 (3)	0.32688 (17)	0.40905 (16)	0.0239 (6)	
H16	0.8781	0.3240	0.3560	0.029*	
C17	0.9710 (3)	0.25142 (17)	0.47177 (16)	0.0233 (6)	
C18	0.9502 (3)	0.33713 (16)	0.55572 (15)	0.0211 (6)	
C19	1.0937 (4)	0.09138 (17)	0.52594 (18)	0.0334 (7)	
H19A	1.1971	0.1126	0.5401	0.050*	
H19B	1.1298	0.0376	0.5074	0.050*	
H19C	1.0038	0.0706	0.5768	0.050*	
C20	1.0601 (3)	0.28177 (16)	0.69881 (16)	0.0221 (6)	
C21	0.9977 (3)	0.09825 (16)	0.86629 (15)	0.0249 (6)	
C22	0.7537 (4)	0.07751 (18)	0.95834 (16)	0.0313 (7)	
H22	0.6371	0.0604	0.9865	0.038*	
C23	0.8294 (4)	0.06179 (17)	0.88397 (16)	0.0259 (6)	
C24	1.1580 (4)	0.17922 (19)	0.94119 (18)	0.0367 (7)	
H24A	1.1204	0.1980	0.9906	0.055*	
H24B	1.1880	0.2365	0.8895	0.055*	
H24C	1.2623	0.1349	0.9519	0.055*	
C25	0.7428 (3)	0.01230 (16)	0.84415 (15)	0.0246 (6)	
C26	0.4366 (4)	−0.14953 (17)	0.85757 (17)	0.0297 (6)	
H26A	0.4199	−0.1609	0.8065	0.044*	
H26B	0.3240	−0.1263	0.8811	0.044*	
H26C	0.4767	−0.2097	0.9009	0.044*	

### Atomic displacement parameters (Å^2^)

**Table:**

	*U*^11^	*U*^22^	*U*^33^	*U*^12^	*U*^13^	*U*^23^
S1	0.0221 (4)	0.0230 (3)	0.0234 (3)	0.0007 (3)	−0.0029 (3)	−0.0116 (3)
S2	0.0239 (4)	0.0192 (3)	0.0317 (4)	0.0031 (3)	−0.0010 (3)	−0.0051 (3)
O1	0.0284 (11)	0.0289 (9)	0.0222 (9)	0.0028 (8)	−0.0049 (8)	−0.0096 (8)
O2	0.0415 (12)	0.0205 (9)	0.0287 (10)	0.0040 (8)	−0.0094 (9)	−0.0113 (8)
O3	0.0347 (11)	0.0193 (9)	0.0233 (9)	0.0069 (8)	−0.0078 (8)	−0.0077 (7)
O4	0.0290 (11)	0.0271 (9)	0.0368 (10)	−0.0005 (8)	−0.0028 (8)	−0.0199 (8)
O5	0.0214 (11)	0.0373 (10)	0.0323 (10)	0.0059 (8)	−0.0076 (8)	−0.0165 (8)
O6	0.0316 (11)	0.0263 (9)	0.0235 (9)	0.0038 (8)	−0.0092 (8)	−0.0121 (7)
O7	0.0377 (12)	0.0265 (9)	0.0370 (11)	0.0034 (8)	−0.0042 (9)	−0.0208 (8)
O8	0.0362 (12)	0.0238 (9)	0.0264 (10)	0.0031 (8)	−0.0085 (8)	−0.0099 (8)
O9	0.0368 (12)	0.0196 (9)	0.0421 (11)	0.0040 (8)	0.0031 (9)	−0.0105 (8)
O10	0.0221 (11)	0.0329 (10)	0.0472 (12)	0.0008 (8)	−0.0071 (9)	−0.0086 (9)
N1	0.0205 (12)	0.0218 (10)	0.0205 (11)	−0.0026 (9)	−0.0011 (9)	−0.0058 (9)
N2	0.0213 (12)	0.0178 (10)	0.0232 (11)	0.0003 (8)	−0.0016 (9)	−0.0066 (9)
N3	0.0238 (12)	0.0198 (10)	0.0197 (11)	0.0026 (8)	−0.0019 (9)	−0.0093 (8)
N4	0.0246 (13)	0.0171 (10)	0.0196 (10)	0.0053 (8)	−0.0032 (9)	−0.0057 (8)
N5	0.0399 (15)	0.0255 (11)	0.0251 (12)	0.0036 (10)	−0.0017 (11)	−0.0037 (10)
N6	0.0538 (19)	0.0303 (13)	0.0362 (14)	−0.0034 (12)	0.0064 (13)	0.0013 (11)
N7	0.0271 (14)	0.0277 (12)	0.0424 (14)	−0.0009 (10)	−0.0050 (11)	−0.0173 (11)
N8	0.0252 (14)	0.0316 (12)	0.0614 (17)	0.0047 (10)	−0.0119 (12)	−0.0288 (12)
N9	0.0380 (17)	0.0719 (19)	0.0605 (19)	0.0015 (14)	−0.0009 (14)	−0.0443 (16)
N10	0.0320 (16)	0.0691 (18)	0.0476 (16)	0.0033 (13)	0.0005 (12)	−0.0354 (14)
N11	0.0210 (12)	0.0222 (10)	0.0233 (11)	−0.0020 (9)	−0.0005 (9)	−0.0102 (9)
N12	0.0188 (12)	0.0194 (10)	0.0277 (12)	−0.0023 (8)	0.0000 (9)	−0.0112 (9)
N13	0.0262 (13)	0.0164 (10)	0.0257 (11)	0.0031 (8)	−0.0074 (9)	−0.0105 (9)
N14	0.0302 (13)	0.0196 (10)	0.0196 (11)	0.0033 (9)	0.0006 (9)	−0.0066 (9)
N15	0.0332 (15)	0.0229 (11)	0.0283 (12)	−0.0032 (9)	−0.0063 (10)	−0.0046 (9)
N16	0.0431 (16)	0.0301 (12)	0.0271 (12)	−0.0043 (11)	0.0035 (11)	−0.0083 (10)
N17	0.0276 (13)	0.0261 (11)	0.0213 (11)	0.0023 (9)	−0.0047 (9)	−0.0103 (9)
N18	0.0244 (13)	0.0236 (11)	0.0244 (12)	0.0058 (9)	−0.0062 (10)	−0.0097 (9)
N19	0.0337 (15)	0.0309 (12)	0.0215 (12)	0.0039 (10)	−0.0019 (10)	−0.0085 (10)
N20	0.0323 (14)	0.0287 (12)	0.0223 (12)	0.0013 (10)	−0.0022 (10)	−0.0055 (9)
C1	0.0329 (17)	0.0333 (14)	0.0283 (14)	−0.0024 (12)	−0.0032 (12)	−0.0171 (12)
C2	0.0183 (14)	0.0257 (13)	0.0189 (13)	−0.0025 (10)	0.0006 (10)	−0.0064 (10)
C3	0.0201 (15)	0.0198 (12)	0.0239 (13)	0.0000 (10)	−0.0025 (11)	−0.0033 (10)
C4	0.0208 (15)	0.0191 (12)	0.0242 (13)	−0.0038 (10)	0.0013 (11)	−0.0057 (10)
C5	0.0153 (14)	0.0200 (12)	0.0221 (13)	−0.0055 (10)	0.0001 (10)	−0.0049 (10)
C6	0.048 (2)	0.0288 (14)	0.0254 (14)	0.0016 (13)	−0.0055 (13)	−0.0130 (12)
C7	0.0187 (14)	0.0172 (12)	0.0224 (13)	−0.0037 (10)	0.0002 (10)	−0.0061 (10)
C8	0.0308 (16)	0.0236 (13)	0.0180 (13)	−0.0003 (11)	−0.0026 (11)	−0.0086 (10)
C9	0.045 (2)	0.0371 (16)	0.0341 (17)	−0.0085 (14)	0.0087 (14)	−0.0040 (13)
C10	0.0319 (17)	0.0302 (14)	0.0207 (13)	−0.0044 (12)	0.0026 (12)	−0.0098 (11)
C11	0.047 (2)	0.0314 (15)	0.0322 (16)	0.0151 (13)	−0.0080 (14)	−0.0075 (13)
C12	0.0226 (16)	0.0330 (14)	0.0294 (15)	−0.0038 (11)	0.0002 (12)	−0.0164 (12)
C13	0.042 (2)	0.0348 (16)	0.085 (3)	0.0064 (14)	−0.0173 (18)	−0.0268 (17)
C14	0.0323 (17)	0.0321 (14)	0.0224 (14)	0.0030 (12)	−0.0082 (12)	−0.0133 (11)
C15	0.0157 (14)	0.0254 (13)	0.0256 (14)	−0.0040 (10)	0.0009 (11)	−0.0119 (11)
C16	0.0229 (15)	0.0280 (13)	0.0238 (13)	−0.0051 (11)	0.0001 (11)	−0.0133 (11)
C17	0.0185 (15)	0.0240 (13)	0.0304 (14)	−0.0047 (10)	0.0028 (11)	−0.0151 (11)
C18	0.0177 (14)	0.0198 (12)	0.0241 (13)	−0.0023 (10)	0.0003 (11)	−0.0074 (10)
C19	0.0363 (18)	0.0193 (13)	0.0460 (17)	0.0026 (11)	−0.0045 (14)	−0.0150 (12)
C20	0.0192 (15)	0.0191 (12)	0.0249 (14)	−0.0002 (10)	0.0002 (11)	−0.0063 (11)
C21	0.0276 (16)	0.0201 (12)	0.0225 (13)	0.0031 (11)	−0.0040 (11)	−0.0040 (10)
C22	0.0332 (18)	0.0277 (14)	0.0291 (15)	−0.0044 (12)	0.0031 (13)	−0.0085 (12)
C23	0.0269 (16)	0.0213 (12)	0.0249 (14)	0.0015 (11)	−0.0010 (11)	−0.0053 (11)
C24	0.0420 (19)	0.0344 (15)	0.0341 (16)	−0.0048 (13)	−0.0132 (14)	−0.0096 (13)
C25	0.0243 (16)	0.0198 (12)	0.0245 (14)	0.0037 (11)	−0.0008 (11)	−0.0050 (11)
C26	0.0312 (17)	0.0281 (14)	0.0329 (15)	−0.0003 (12)	−0.0039 (12)	−0.0154 (12)

### Geometric parameters (Å, º)

**Table:**

S1—O5	1.4212 (18)	N15—C21	1.364 (3)
S1—O4	1.4238 (16)	N15—C24	1.467 (4)
S1—N4	1.647 (2)	N16—C22	1.336 (4)
S1—C8	1.745 (3)	N17—N18	1.327 (3)
S2—O10	1.420 (2)	N17—C25	1.336 (3)
S2—O9	1.4267 (18)	N18—N19	1.323 (3)
S2—N14	1.6431 (19)	N18—C26	1.451 (3)
S2—C21	1.747 (3)	N19—N20	1.324 (3)
O1—C2	1.333 (3)	N20—C25	1.362 (3)
O1—C1	1.443 (3)	C1—H1A	0.9800
O2—C4	1.342 (3)	C1—H1B	0.9800
O2—C6	1.441 (3)	C1—H1C	0.9800
O3—C7	1.222 (3)	C2—C3	1.387 (3)
O6—C15	1.340 (3)	C3—C4	1.373 (3)
O6—C14	1.440 (3)	C3—H3	0.9500
O7—C17	1.349 (3)	C6—H6A	0.9800
O7—C19	1.447 (3)	C6—H6B	0.9800
O8—C20	1.211 (3)	C6—H6C	0.9800
N1—C5	1.323 (3)	C8—C10	1.400 (4)
N1—C2	1.338 (3)	C9—C10	1.398 (4)
N2—C5	1.338 (3)	C9—H9	0.9500
N2—C4	1.345 (3)	C10—C12	1.455 (3)
N3—C7	1.380 (3)	C11—H11A	0.9800
N3—C5	1.399 (3)	C11—H11B	0.9800
N3—H3N	0.8800	C11—H11C	0.9800
N4—C7	1.370 (3)	C13—H13A	0.9800
N4—H4N	0.8800	C13—H13B	0.9800
N5—N6	1.341 (3)	C13—H13C	0.9800
N5—C8	1.361 (3)	C14—H14A	0.9800
N5—C11	1.465 (3)	C14—H14B	0.9800
N6—C9	1.324 (4)	C14—H14C	0.9800
N7—C12	1.328 (3)	C15—C16	1.384 (3)
N7—N8	1.344 (3)	C16—C17	1.382 (3)
N8—N9	1.310 (3)	C16—H16	0.9500
N8—C13	1.458 (4)	C19—H19A	0.9800
N9—N10	1.312 (3)	C19—H19B	0.9800
N10—C12	1.350 (3)	C19—H19C	0.9800
N11—C18	1.333 (3)	C21—C23	1.393 (4)
N11—C15	1.345 (3)	C22—C23	1.400 (3)
N12—C17	1.326 (3)	C22—H22	0.9500
N12—C18	1.338 (3)	C23—C25	1.449 (4)
N13—C18	1.376 (3)	C24—H24A	0.9800
N13—C20	1.384 (3)	C24—H24B	0.9800
N13—H13N	0.8800	C24—H24C	0.9800
N14—C20	1.380 (3)	C26—H26A	0.9800
N14—H14N	0.8800	C26—H26B	0.9800
N15—N16	1.343 (3)	C26—H26C	0.9800
			
O5—S1—O4	120.08 (11)	O3—C7—N3	121.2 (2)
O5—S1—N4	110.06 (10)	N4—C7—N3	116.2 (2)
O4—S1—N4	103.25 (10)	N5—C8—C10	107.4 (2)
O5—S1—C8	108.81 (11)	N5—C8—S1	124.3 (2)
O4—S1—C8	107.74 (11)	C10—C8—S1	128.17 (18)
N4—S1—C8	105.99 (11)	N6—C9—C10	112.4 (3)
O10—S2—O9	119.77 (11)	N6—C9—H9	123.8
O10—S2—N14	110.51 (11)	C10—C9—H9	123.8
O9—S2—N14	103.83 (11)	C9—C10—C8	103.2 (2)
O10—S2—C21	108.05 (13)	C9—C10—C12	124.8 (3)
O9—S2—C21	109.42 (12)	C8—C10—C12	132.0 (2)
N14—S2—C21	104.16 (11)	N5—C11—H11A	109.5
C2—O1—C1	117.82 (19)	N5—C11—H11B	109.5
C4—O2—C6	117.94 (19)	H11A—C11—H11B	109.5
C15—O6—C14	117.12 (18)	N5—C11—H11C	109.5
C17—O7—C19	116.6 (2)	H11A—C11—H11C	109.5
C5—N1—C2	114.6 (2)	H11B—C11—H11C	109.5
C5—N2—C4	114.8 (2)	N7—C12—N10	112.7 (2)
C7—N3—C5	129.9 (2)	N7—C12—C10	126.6 (2)
C7—N3—H3N	115.0	N10—C12—C10	120.5 (2)
C5—N3—H3N	115.0	N8—C13—H13A	109.5
C7—N4—S1	125.14 (16)	N8—C13—H13B	109.5
C7—N4—H4N	117.4	H13A—C13—H13B	109.5
S1—N4—H4N	117.4	N8—C13—H13C	109.5
N6—N5—C8	111.0 (2)	H13A—C13—H13C	109.5
N6—N5—C11	118.1 (2)	H13B—C13—H13C	109.5
C8—N5—C11	130.9 (2)	O6—C14—H14A	109.5
C9—N6—N5	105.9 (2)	O6—C14—H14B	109.5
C12—N7—N8	100.5 (2)	H14A—C14—H14B	109.5
N9—N8—N7	114.3 (2)	O6—C14—H14C	109.5
N9—N8—C13	122.4 (2)	H14A—C14—H14C	109.5
N7—N8—C13	123.2 (2)	H14B—C14—H14C	109.5
N8—N9—N10	106.2 (2)	O6—C15—N11	112.2 (2)
N9—N10—C12	106.2 (2)	O6—C15—C16	124.5 (2)
C18—N11—C15	115.0 (2)	N11—C15—C16	123.3 (2)
C17—N12—C18	115.4 (2)	C17—C16—C15	115.3 (2)
C18—N13—C20	130.6 (2)	C17—C16—H16	122.3
C18—N13—H13N	114.7	C15—C16—H16	122.3
C20—N13—H13N	114.7	N12—C17—O7	118.0 (2)
C20—N14—S2	122.10 (18)	N12—C17—C16	123.7 (2)
C20—N14—H14N	119.0	O7—C17—C16	118.3 (2)
S2—N14—H14N	119.0	N11—C18—N12	127.2 (2)
N16—N15—C21	111.3 (2)	N11—C18—N13	114.6 (2)
N16—N15—C24	117.3 (2)	N12—C18—N13	118.2 (2)
C21—N15—C24	131.4 (2)	O7—C19—H19A	109.5
C22—N16—N15	105.5 (2)	O7—C19—H19B	109.5
N18—N17—C25	102.1 (2)	H19A—C19—H19B	109.5
N19—N18—N17	113.8 (2)	O7—C19—H19C	109.5
N19—N18—C26	124.1 (2)	H19A—C19—H19C	109.5
N17—N18—C26	122.0 (2)	H19B—C19—H19C	109.5
N18—N19—N20	106.5 (2)	O8—C20—N14	122.8 (2)
N19—N20—C25	105.9 (2)	O8—C20—N13	120.8 (2)
O1—C1—H1A	109.5	N14—C20—N13	116.4 (2)
O1—C1—H1B	109.5	N15—C21—C23	107.4 (2)
H1A—C1—H1B	109.5	N15—C21—S2	123.5 (2)
O1—C1—H1C	109.5	C23—C21—S2	129.0 (2)
H1A—C1—H1C	109.5	N16—C22—C23	112.2 (3)
H1B—C1—H1C	109.5	N16—C22—H22	123.9
O1—C2—N1	119.3 (2)	C23—C22—H22	123.9
O1—C2—C3	117.0 (2)	C21—C23—C22	103.6 (2)
N1—C2—C3	123.7 (2)	C21—C23—C25	132.5 (2)
C4—C3—C2	115.6 (2)	C22—C23—C25	123.8 (2)
C4—C3—H3	122.2	N15—C24—H24A	109.5
C2—C3—H3	122.2	N15—C24—H24B	109.5
O2—C4—N2	118.3 (2)	H24A—C24—H24B	109.5
O2—C4—C3	118.6 (2)	N15—C24—H24C	109.5
N2—C4—C3	123.1 (2)	H24A—C24—H24C	109.5
N1—C5—N2	128.1 (2)	H24B—C24—H24C	109.5
N1—C5—N3	114.3 (2)	N17—C25—N20	111.8 (2)
N2—C5—N3	117.5 (2)	N17—C25—C23	119.4 (2)
O2—C6—H6A	109.5	N20—C25—C23	128.8 (2)
O2—C6—H6B	109.5	N18—C26—H26A	109.5
H6A—C6—H6B	109.5	N18—C26—H26B	109.5
O2—C6—H6C	109.5	H26A—C26—H26B	109.5
H6A—C6—H6C	109.5	N18—C26—H26C	109.5
H6B—C6—H6C	109.5	H26A—C26—H26C	109.5
O3—C7—N4	122.6 (2)	H26B—C26—H26C	109.5
			
O5—S1—N4—C7	−46.2 (2)	N5—C8—C10—C12	179.0 (3)
O4—S1—N4—C7	−175.6 (2)	S1—C8—C10—C12	−5.2 (5)
C8—S1—N4—C7	71.3 (2)	N8—N7—C12—N10	−0.2 (3)
C8—N5—N6—C9	−0.8 (3)	N8—N7—C12—C10	−176.4 (3)
C11—N5—N6—C9	−179.6 (3)	N9—N10—C12—N7	0.2 (3)
C12—N7—N8—N9	0.2 (3)	N9—N10—C12—C10	176.7 (3)
C12—N7—N8—C13	178.5 (3)	C9—C10—C12—N7	140.6 (3)
N7—N8—N9—N10	−0.1 (3)	C8—C10—C12—N7	−39.5 (5)
C13—N8—N9—N10	−178.4 (3)	C9—C10—C12—N10	−35.4 (4)
N8—N9—N10—C12	−0.1 (3)	C8—C10—C12—N10	144.6 (3)
O10—S2—N14—C20	−62.4 (2)	C14—O6—C15—N11	174.7 (2)
O9—S2—N14—C20	167.96 (19)	C14—O6—C15—C16	−5.5 (3)
C21—S2—N14—C20	53.4 (2)	C18—N11—C15—O6	−179.3 (2)
C21—N15—N16—C22	0.2 (3)	C18—N11—C15—C16	0.9 (3)
C24—N15—N16—C22	178.3 (2)	O6—C15—C16—C17	−177.9 (2)
C25—N17—N18—N19	0.3 (2)	N11—C15—C16—C17	1.9 (4)
C25—N17—N18—C26	177.8 (2)	C18—N12—C17—O7	−179.8 (2)
N17—N18—N19—N20	−0.2 (3)	C18—N12—C17—C16	1.6 (3)
C26—N18—N19—N20	−177.7 (2)	C19—O7—C17—N12	3.8 (3)
N18—N19—N20—C25	0.0 (2)	C19—O7—C17—C16	−177.5 (2)
C1—O1—C2—N1	7.6 (3)	C15—C16—C17—N12	−3.2 (4)
C1—O1—C2—C3	−174.1 (2)	C15—C16—C17—O7	178.2 (2)
C5—N1—C2—O1	175.3 (2)	C15—N11—C18—N12	−2.9 (4)
C5—N1—C2—C3	−2.9 (3)	C15—N11—C18—N13	176.3 (2)
O1—C2—C3—C4	−174.8 (2)	C17—N12—C18—N11	1.7 (4)
N1—C2—C3—C4	3.4 (4)	C17—N12—C18—N13	−177.5 (2)
C6—O2—C4—N2	−1.9 (3)	C20—N13—C18—N11	176.5 (2)
C6—O2—C4—C3	178.0 (2)	C20—N13—C18—N12	−4.2 (4)
C5—N2—C4—O2	179.3 (2)	S2—N14—C20—O8	7.5 (3)
C5—N2—C4—C3	−0.6 (3)	S2—N14—C20—N13	−171.02 (17)
C2—C3—C4—O2	178.6 (2)	C18—N13—C20—O8	179.7 (2)
C2—C3—C4—N2	−1.5 (4)	C18—N13—C20—N14	−1.8 (4)
C2—N1—C5—N2	0.5 (4)	N16—N15—C21—C23	−0.4 (3)
C2—N1—C5—N3	−178.47 (19)	C24—N15—C21—C23	−178.2 (2)
C4—N2—C5—N1	1.2 (4)	N16—N15—C21—S2	−179.42 (17)
C4—N2—C5—N3	−179.9 (2)	C24—N15—C21—S2	2.8 (4)
C7—N3—C5—N1	171.6 (2)	O10—S2—C21—N15	13.3 (2)
C7—N3—C5—N2	−7.5 (4)	O9—S2—C21—N15	145.26 (19)
S1—N4—C7—O3	−0.4 (4)	N14—S2—C21—N15	−104.2 (2)
S1—N4—C7—N3	−178.80 (17)	O10—S2—C21—C23	−165.5 (2)
C5—N3—C7—O3	−177.1 (2)	O9—S2—C21—C23	−33.5 (3)
C5—N3—C7—N4	1.4 (4)	N14—S2—C21—C23	77.0 (2)
N6—N5—C8—C10	1.2 (3)	N15—N16—C22—C23	0.1 (3)
C11—N5—C8—C10	179.8 (3)	N15—C21—C23—C22	0.4 (3)
N6—N5—C8—S1	−174.8 (2)	S2—C21—C23—C22	179.38 (19)
C11—N5—C8—S1	3.8 (4)	N15—C21—C23—C25	−175.6 (2)
O5—S1—C8—N5	16.9 (3)	S2—C21—C23—C25	3.4 (4)
O4—S1—C8—N5	148.5 (2)	N16—C22—C23—C21	−0.3 (3)
N4—S1—C8—N5	−101.5 (2)	N16—C22—C23—C25	176.1 (2)
O5—S1—C8—C10	−158.3 (2)	N18—N17—C25—N20	−0.2 (3)
O4—S1—C8—C10	−26.6 (3)	N18—N17—C25—C23	179.0 (2)
N4—S1—C8—C10	83.4 (3)	N19—N20—C25—N17	0.1 (3)
N5—N6—C9—C10	0.1 (3)	N19—N20—C25—C23	−179.0 (2)
N6—C9—C10—C8	0.5 (3)	C21—C23—C25—N17	151.6 (3)
N6—C9—C10—C12	−179.5 (3)	C22—C23—C25—N17	−23.7 (4)
N5—C8—C10—C9	−1.0 (3)	C21—C23—C25—N20	−29.3 (4)
S1—C8—C10—C9	174.8 (2)	C22—C23—C25—N20	155.4 (2)

### Hydrogen-bond geometry (Å, º)

**Table:**

*D*—H···*A*	*D*—H	H···*A*	*D*···*A*	*D*—H···*A*
N3—H3*N*···N11^i^	0.88	2.65	3.494 (3)	162
N4—H4*N*···N2	0.88	1.89	2.611 (3)	138
N13—H13*N*···O3^i^	0.88	2.04	2.844 (2)	151
N14—H14*N*···N12	0.88	1.95	2.636 (3)	134
C1—H1*C*···O8^ii^	0.98	2.58	3.559 (3)	178
C11—H11*B*···O3	0.98	2.38	3.236 (3)	146
C11—H11*B*···O8^i^	0.98	2.57	3.278 (3)	129
C11—H11*C*···N10^iii^	0.98	2.58	3.290 (4)	130
C13—H13*A*···O9^iv^	0.98	2.40	3.374 (4)	175
C14—H14*A*···O5^v^	0.98	2.39	3.335 (3)	162
C22—H22···N17^vi^	0.95	2.57	3.435 (3)	152
C24—H24*B*···O8	0.98	2.33	3.080 (3)	133
C26—H26*B*···N16^vi^	0.98	2.52	3.376 (3)	146

Symmetry codes: (i) −*x*+1, −*y*+1, −*z*+1; (ii) *x*−1, *y*, *z*; (iii) −*x*+1, −*y*+1, −*z*; (iv) −*x*+2, −*y*, −*z*+1; (v) *x*+1, *y*, *z*; (vi) −*x*+1, −*y*, −*z*+2.
